# Determinants and clinical outcomes of stroke following revascularization among patients with reduced ejection fraction

**DOI:** 10.1002/brb3.2927

**Published:** 2023-03-01

**Authors:** Shaoping Wang, Yuhua Ran, Shujuan Cheng, Yi Lyu, Jinghua Liu

**Affiliations:** ^1^ Department of Cardiology, Beijing Anzhen Hospital Capital Medical University, Beijing Institute of Heart Lung and Blood Vessel Diseases Beijing China; ^2^ Department of Neuropsychopharmacology Beijing Institute of Toxicology and Pharmacology Beijing China; ^3^ Department of Anesthesiology, Minhang Hospital Fudan University Shanghai China

**Keywords:** ejection fraction, heart failure, revascularization, stroke

## Abstract

**Objective:**

Stoke after revascularization including both percutaneous coronary intervention (PCI) and coronary artery bypass grafting (CABG) is an uncommon but devastating complication. Patients with reduced ejection fraction (EF) had an increased risk of stroke after revascularization. However, little is known about the determinants and outcomes of stroke among patients with reduced EF following revascularization.

**Materials and Methods:**

A cohort study of patients with preoperative reduced EF (≤40%) who received revascularization by either PCI or CABG between January 1, 2005 and December 31, 2014 was performed. Multivariate logistic regression was used to identify independent correlates of stroke. Logistic regression models were applied to evaluate the association of stroke with clinical outcomes.

**Results:**

A total of 1937 patients were enrolled in this study. Of these, 111 (5.7%) patients suffered from stroke during the median 3.5‐year follow‐up. Older age (odds ratio [OR], 1.03; 95% CI, 1.01–1.05; *p* = .009), history of hypertension (OR, 1.79; 95% CI, 1.18–2.73; *p* = .007), and history of stroke (OR, 2.00; 95% CI, 1.19–3.36; *p* = .008) were found to be independent predictors for stroke. Patients with and without stroke had similar risk of all‐cause death (OR, 0.91; 95% CI, 0.59–1.41; *p* = .670). However, stroke was associated with higher odds ratio of heart failure (HF) hospitalization (OR, 2.77; 95% CI, 1.74–4.40; *p* < .001) and composite end point (OR, 1.61; 95% CI, 1.07–2.42; *p* = .021).

**Conclusions:**

Further research appears warranted to minimize the complication of stroke and improve long‐term outcomes among patients with reduced EF who underwent such high risk revascularization procedural.

## Graphical abstract

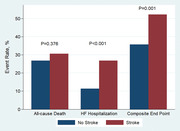

## 1 INTRODUCTION

Stoke after revascularization including both percutaneous coronary intervention (PCI) and coronary artery bypass grafting (CABG) is an uncommon but devastating complication (Gaudino et al., 2019). According to the Society of Thoracic Surgeons CABG Adult Cardiac Surgery Databases, the incidence of stroke after CABG is 1.3% (ElBardissi et al., 2012). The perioperative mortality for patients who had a stroke after CABG is 16.4% (Edwards et al., 2016). In large observational PCI studies, the risk of stoke ranged between 0.2% and 0.5% and the in‐hospital morality was from 19.2% to 30% (Aggarwal et al., 2009; Kwok et al., 2015; Moreyra et al., 2017; Werner et al., 2013). Thus better understanding of the determinants of stroke after revascularization has important implications for the management of patients with high risk.

For patients with coronary artery disease (CAD) and heart failure (HF), revascularization by either PCI (Kunadian et al., 2012; Lee et al., 2012; Yang et al., 2013) or CABG (Velazquez et al., 2016) may improve long‐term outcome by attenuating the ischemic state and reversing left ventricular (LV) remodeling (Adachi et al., 2016; Michler et al., 2013; Vakil et al., 2016). However, HF had been found to be an independent predictor of stroke after revascularization (Charlesworth et al., 2003; Tian et al., 2017; Werner et al., 2013). Patients with congestive HF had a 1.62‐fold increased risk of stroke after PCI in acute coronary syndrome (Werner et al., 2013). Patients with reduced ejection fraction (EF < 40%) had about a 1.5‐fold increased odds of perioperative stroke following isolated CABG (Charlesworth et al., 2003). Procedure complication of stroke may partially obviate the benefit of revascularization among patients with reduced EF. Unfortunately, little is known about the incidence and determinants of stroke among patients with reduced EF following either PCI or CABG so far.

Therefore, this study was performed to demonstrate (1) the determinants of stroke following revascularization among patients with preoperative EF ≤ 40% and (2) the difference of clinical outcomes between patients with and without stroke after revascularization in a large real‐world clinical cohort.

## 2 METHODS

### 2.1 Patient selection

This was a real‐world cohort study that used data from Beijing Anzhen Hospital, which is a large referral hospital in China that focuses on heart, lung, and blood vessel diseases. The study was registered in Chinese Clinical Trial Registry (No. ChiCTR2100044378). The study protocol was approved by the hospital's ethics committee.

CAD patients with reduced EF (≤40%) who underwent CABG or PCI with a drug‐eluting stent between January 2005 and December 2014 were enrolled. Patients were excluded if they had concomitant noncoronary surgery, were diagnosed as ST‐segment elevation myocardial infarction (MI), and had no angiography record.

### 2.2 Data collection and definitions

Baseline demographic, clinical, laboratory, and angiographic parameters for the study patients were ascertained from Beijing Anzhen Hospital medical records. Baseline EF was captured within 30 days before PCI or CABG. Complete revascularization was defined as successful PCI (residual stenosis of < 30%) of all angiographically significant lesions (≥70% diameter stenosis) in three coronary arteries and their major branches. A staged procedure within 90 days after discharge was acceptable. For CABG, grafting of every primary coronary artery with ≥70% diameter stenosis was accepted as complete revascularization.

Outcome data were obtained from medical records at Beijing Anzhen Hospital and through telephone follow‐up. Stroke was defined as a persistent loss of neurological function caused by a cerebral ischemic or hemorrhagic event. HF hospitalization was defined as the first readmission with a primary diagnosis of HF after discharge from the index procedure. We also assessed composite end point as all‐cause death and HF hospitalization.

### 2.3 Statistical analysis

Continuous variables were expressed as mean (SD) and categorical variables as counts (percentages). Highly skewed continuous distributions were described by median (interquartile range). Baseline characteristics were compared between patients with and without stroke by using a student t test, rank sum test, or χ^2^ test, as appropriate. Multivariate logistic regression was used to identify independent correlates of stroke. Variables of demographics and history, preoperative echocardiography values, angiography and therapy (PCI or CABG) as well as medications at discharge were included in the analysis. All variables that had marginal association in univariate analysis (*p* < 0.100) were adopted as independent variables in multivariate logistic regression analysis. Logistic regression models were applied to evaluate the association of stroke with clinical outcomes. Variables of age, sex, current smoking status, history of hypertension, diabetes mellitus, cerebral vascular disease and myocardial infarction, preoperative ejection fraction, coronary multivessel disease, and treatment of PCI or CABG were included in adjusted model. All statistical analyses were based on two‐tailed tests. *p* < .05 was considered statistically significant. Statistical analyses were performed with Stata version 14.0 (StataCorp).

## 3 RESULTS

### 3.1 Baseline characteristics

Among 2380 initially identified patients, 306 CABG patients had concomitant noncoronary surgery, 102 patients were diagnosed as ST‐segment elevation MI, 35 patients were further excluded because of no angiography record.

This study cohort finally included 1937 patients who had a preoperative EF ≤40% and underwent either PCI (45.4%) or CABG (54.6%) (Table 1). Of these, 111 (5.7%) patients suffered from stroke during the median 3.5‐year follow‐up. Mean age in the stroke group was significantly higher than in the group without stroke (69.4 ± 9.2 vs. 66.1 ± 10.6; *p* = .001). Sex distribution was similar between two groups. The stroke group had a significantly higher prevalence of hypertension (67.6% vs. 52.7%; *p* = .002) and history of stroke (18.9% vs. 8.8%; *p* < .001). The stroke group had a significant lower estimated glomerular filtration rate (eGFR) (78.3 ± 21.6 vs. 83.4 ± 24.4; *p* = .032). The mean (SD) preoperative EF was 36.1 (4.4). There was no significantly difference of preoperative EF between two groups. The stroke group had significantly higher proportion undergoing revascularization by CABG (68.5% vs. 53.8%; *p* = .003) compared to the group without stroke. The stroke group tended to have higher prevalence of multivessel disease (88.3% vs. 81.7%; *p* = .079) and percentages of complete revascularization (64.9% vs. 56.7%; *p* = .091). The percentages of prescription of aspirin at discharge between two groups were similar. However, the stroke group had significantly lower percentage of prescription of clopidogrel or ticagrelor (55.0% vs. 67.6%; *p* = .006). The stroke group tended to have lower percentage of prescription of statin (62.6% vs. 69.8%; *p* = .092) compared to group without stroke.

**TABLE 1:** Patient characteristics at baseline (with versus without stroke)

Characteristic	All patients (*N* = 1937)	Stroke (*n* = 111)	No stroke (*n* = 1826)	*p* Value
Demographics and history				
Age, years	66.3 (10.6)	69.4 (9.2)	66.1 (10.6)	.001
Men	1603 (82.8)	94 (84.7)	1509 (82.6)	.580
Weight, kg	71.3 (11.4)	72.7 (11.0)	71.2 (11.5)	.164
Current smoker	633 (32.7)	38 (34.2)	595 (32.6)	.719
Hypertension	1038 (53.6)	75 (67.6)	963 (52.7)	.002
DM	686 (35.4)	43 (38.7)	643 (35.2)	.451
Insulin‐dependent DM	145 (7.6)	11 (10.1)	134 (7.4)	.308
eGFR	83.1 (24.3)	78.3 (21.6)	83.4 (24.4)	.032
LDL‐C, mmol/L	2.7 (0.9)	2.8 (1.0)	2.7 (0.9)	.289
History of stroke	182 (9.4)	21 (18.9)	161 (8.8)	<.001
Atrial fibrillation	98 (5.1)	6 (5.4)	92 (5.0)	.864
History of MI	962 (49.7)	50 (45.1)	912 (50.0)	.316
History of PCI	304 (15.7)	14 (12.6)	290 (15.9)	.358
History of CABG	33 (1.7)	3 (2.7)	30 (1.6)	.402
Echocardiography				
Preoperative				
EF, %	36.1 (4.4)	36.0 (4.7)	36.1 (4.4)	.934
LVEDD, mm	58.8 (7.1)	59.2 (6.6)	58.7 (7.1)	.558
LVESD, mm	46.1 (8.0)	46.7 (7.3)	46.0 (8.0)	.367
MR (moderate or severe)	306 (15.8)	13 (11.7)	293 (16.1)	.224
Angiography and therapy				
Multivessel disease	1590 (82.1)	98 (88.3)	1492 (81.7)	.079
Left main disease	135 (7.0)	9 (8.1)	126 (6.9)	.628
PCI	879 (45.4)	35 (31.5)	844 (46.2)	.003
CABG	1058 (54.6)	76 (68.5)	982 (53.8)	.003
Complete revascularization	1107 (57.2)	72 (64.9)	1035 (56.7)	.091
Medications at discharge				
Aspirin	1747 (92.4)	106 (95.5)	1641 (92.2)	.209
Clopidogrel/ticagrelor	1263 (66.8)	61 (55.0)	1202 (67.6)	.006
Statin	1310 (69.3)	69 (62.2)	1241 (69.8)	.092
ACEi or ARB	831(44.0)	47(42.3)	784(44.1)	.722
β‐Blocker	1465 (77.5)	84 (75.7)	1381 (77.6)	.633
MRA	315 (16.7)	19 (17.1)	296 (16.6)	.896
Diuretics	600 (31.8)	31 (27.9)	569 (32.0)	.373
Digoxin	329 (17.4)	17 (15.3)	312 (17.5)	.549

Abbreviations: ACEi, angiotensin‐converting enzyme inhibitor; ARB, angiotensin receptor blocker; CABG, coronary artery bypass grafting; DM, diabetes mellitus; EF, ejection fraction; eGFR, estimated glomerular filtration rate; LDL‐C, low‐density lipoprotein cholesterol; LVEDD, left ventricular end‐diastolic diameter; LVESD, left ventricular end‐systolic diameter; MI, myocardial infarction; MR, mitral regurgitation; MRA, mineralocorticoid receptor antagonist; PCI, percutaneous coronary intervention.

^a^Values are mean (SD) or no. of patients (%).

### 3.2 Predictors of stroke

Evaluation for independent predictor of stroke showed that older patients had greater odds of being in the stroke group (odds ratio [OR], 1.03; 95% CI, 1.01–1.05; *p* = .009) (Table 2). History of hypertension (OR, 1.79; 95% CI, 1.18–2.73; *p* = .007) and history of stroke (OR, 2.00; 95% CI, 1.19–3.36; *p* = .008) were also found to be independent predictors for stroke. Although compared to PCI, treatment of CABG was significantly associated with stroke (OR, 1.87; 95% CI, 1.24–2.81; *p* = .003) in the univariate analysis, CABG was not an independent correlate of stroke (OR, 1.49; 95% CI, 0.81–2.73; *p* = .198) in the multivariate analysis. Neither anatomic severity of coronary vessels (as indicated by multivessel disease and left main disease) nor extent of revascularization (complete vs. incomplete) was an independent correlate of stroke. No medication at discharge including aspirin, clopidogrel, and statin was found to be an independent correlate of stroke in the multivariate model.

**TABLE 2:** Baseline factors associated with stroke in a multivariate model

Variables	Univariate analysis		Multivariate analysis	
	OR (95% CI)	*p* Value	OR (95% CI)	*p* Value
Age	1.03 (1.01–1.05)	.001	1.03 (1.01–1.05)	.009
Male sex	1.16 (0.68–1.97)	.580		
Weight	1.01 (1.00–1.03)	.163		
Current smoker	1.08 (0.72–1.61)	.719		
Hypertension	1.87 (1.24–2.81)	.003	1.79 (1.18–2.73)	.007
Diabetes	1.16 (0.78–1.72)	.451		
eGFR	0.99 (0.98–1.00)	.028	1.00 (0.99–1.01)	.400
LDL‐C, mmol/L	1.11 (0.91–1.35)	.289		
History of stroke	2.41 (1.46–3.99)	.001	2.00 (1.19–3.36)	.008
History of MI	0.82 (0.56–1.21)	.317		
Atrial fibrillation	1.08 (0.46–2.52)	.864		
History of PCI	0.76 (0.43–1.36)	.359		
Preoperative EF	1.00 (0.96–1.04)	.934		
MR (moderate or severe)	0.69 (0.38–1.25)	.227		
Multivessel disease	1.69 (0.93–3.05)	.082	1.32 (0.67–2.62)	.426
Left main disease	1.19 (0.59–2.41)	.628		
CABG*	1.87 (1.24–2.81)	.003	1.49 (0.81–2.73)	.198
Complete revascularization	1.41 (0.95–2.11)	.092	1.41 (0.89–2.26)	.145
Aspirin	1.78 (0.72–4.45)	.215		
Clopidogrel/ticagrelor	0.59 (0.40–0.86)	.007	0.85 (0.51–1.41)	.528
Statin	0.71 (0.48–1.06)	.094	1.01 (0.64–1.61)	.954
ACEi or ARB	0.93 (0.63–1.37)	.722		
β‐Blocker	0.90 (0.57–1.40)	.633		
MRA	1.03 (0.62–1.72)	.896		
Diuretics	0.82 (0.54–1.26)	.374		
Digoxin	0.85 (0.50–1.45)	.549		

*PCI was set as reference to CABG.

Abbreviations: ACEi, angiotensin‐converting enzyme inhibitor; ARB, angiotensin receptor blocker; DM, diabetes mellitus; EF, ejection fraction; eGFR, estimated glomerular filtration rate; MI, myocardial infarction; MR, mitral regurgitation; MRA, mineralocorticoid receptor antagonist; PCI, percutaneous coronary intervention.

### 3.3 Outcomes

**FIGURE 1:**
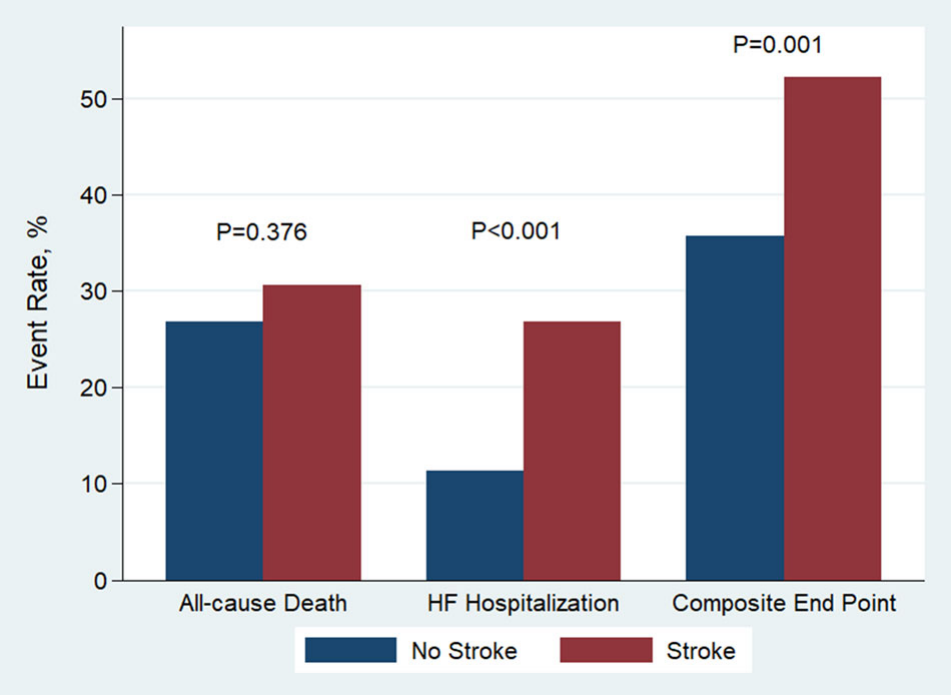
Outcomes comparison between patients with stroke and patients without stroke. HF: heart failure.

The median follow‐up time was 3.5 years, during which 523 patients died and 222 patients had HF hospitalization. Patients with and without stroke had similar risk of all‐cause death both in unadjusted (OR, 1.21; 95% CI, 0.80–1.83; *p* = .376) and adjusted model (OR, 0.91; 95% CI, 0.59–1.41; *p* = .670) (Table 3, Figure 1). However, stroke was associated with higher odds ratio of HF hospitalization (OR, 2.77; 95% CI, 1.74–4.40; *p* < .001) and composite end point (OR, 1.61; 95% CI, 1.07–2.42; *p* = .021).

**TABLE 3:** Risk of outcomes (with versus without stroke)

Outcomes	No. of events	Event rate (%)	Unadjusted		Adjusted^a^	
			OR (95% CI)	*p* Value	OR (95% CI)	*p* Value
All‐cause death						
Stroke	34	30.63	1.21 (0.80–1.83)	.376	0.91 (0.59–1.41)	.670
No stroke	489	26.78	Ref		Ref	
HF hospitalization						
Stroke	29	26.85	2.85 (1.82–4.48)	<.001	2.77 (1.74–4.40)	<.001
No stroke	193	11.41	Ref		Ref	
Composite end point^b^						
Stroke	57	52.29	1.97 (1.34–2.91)	.001	1.61 (1.07–2.42)	.021
No stroke	638	35.70	Ref		Ref	

Abbreviations: DM, diabetes mellitus; HR, hazard ratio; Ref, reference.

^a^ Variables of age, sex, current smoking status, history of (hypertension, diabetes mellitus, cerebral vascular disease, and myocardial infarction), preoperative ejection fraction, coronary multivessel disease, and treatment of PCI or CABG were included in adjusted model.

^b^ All‐cause death or heart failure hospitalization.

### 3.4 Sensitivity analysis

For PCI cohort, all patients underwent local anesthesia. For CABG cohort, 68 (6.4%) had on‐pump CABG and 990 (93.6%) had off‐pump CABG. The incidence of stroke was similar between on‐pump and off‐pump groups (7.4% vs. 7.2%; *p* = 0.955).

## 4 DISCUSSION

Predictors of stroke after revascularization by PCI or CABG have been determined in prior studies among general patient cohorts. For example, older age, dialysis dependency, severe chronic lung disease, emergency surgery, and atherosclerotic burden in the coronary artery and other vascular beds were the most important risk factors for stroke following CABG (O'Brien et al., 2018; Shahian et al., 2009). Older age, history of prior stroke, acute coronary syndrome, and use of an intraaortic balloon pump were independent predictors of periprocedural stroke among patients treated with PCI (Aggarwal et al., 2009; Kwok et al., 2015; Moreyra et al., 2017; Werner et al., 2013). In addition, CHA2DS2‐VASc score was found to be an independent predictor of the development of post‐procedural ischemic stroke in patients undergoing CABG and PCI (Tian et al., 2017). In the current study, among patients with reduced EF, older age, history of hypertension and history of stroke were found to be independent correlates of stroke following either PCI or CABG. CABG was significantly associated with stroke in the univariate analysis, but it failed to be significant in the multivariate analysis. In the studies comparing CABG with PCI in patients with LV dysfunction, CABG was associated with higher risk of stroke in short‐term (Bangalore et al., 2016; Sun et al., 2020). However, in long‐term analysis, some studies indicated CABG was still associated with higher risk of stroke (Bangalore et al., 2016; Sun et al., 2020), while others demonstrated similar risk between two treatments (Kang et al., 2017; Marui et al., 2015; Marui et al., 2014). In addition, in current study, patients with on‐pump CABG and off‐pump CABG had similar incidence of stroke during follow‐up. The relationship between CABG versus PCI, on‐pump versus off‐pump and risk of stroke needs to be further investigated among patients with reduced EF.

Among CAD patients with multivessel and left main diseases, patients who experienced a stroke within 30 days of the procedure had significantly higher 5‐year mortality versus those without a stroke, both after PCI and CABG (Head et al., 2018). In the current study, among patients with reduced EF, patients with and without stroke had similar risk of all‐cause death. More comorbidity such as severe LV dysfunction, renal dysfunction and ventricular arrhythmia was involved in the death among patients with reduced EF. Thus the relationship between stroke and death might be attenuated. However, stroke was associated with higher odds ratio of HF hospitalization. The potential pathogenesis and clinical implication need to be further investigated.

This was a nonrandomized, observational study from a single center. Therefore, as with any observational study, its generalizability may be limited due to its selection biases. Data of stroke time after revascularization was not available. So the incidence of periprocedural and long‐term stroke and their clinical implications cannot be analyzed. Ischemic and hemorrhagic stroke following revascularization had different pathogenesis (Gaudino et al., 2019) and might have various predictors as well as clinical outcome association, which needs further investigation.

## 5 CONCLUSION

Among patients with reduced EF who underwent revascularization by either PCI or CABG, older age, hypertension, and history of stroke were three independent predictors of stroke after revascularization. Patients with stroke were associated with adverse long‐term outcome of HF hospitalization after revascularization. Further research appears warranted to minimize the complication of stroke and improve long‐term outcomes among patients with LV dysfunction who underwent such high risk revascularization procedural.

## CONFLICT OF INTEREST STATEMENT

The authors declare no conflicts of interests.

### ETHICS STATEMENT

The study protocol was approved by the ethics committee of Beijing Anzhen Hospital (2021004X).

### INFORMED CONSENT

Because this was a retrospective cohort study, written informed consent from the patients was waived. The study conforms with World Medical Association Declaration of Helsinki.

### PEER REVIEW

The peer review history for this article is available at https://publons.com/publon/10.1002/brb3.2927.

## Data Availability

Data are available from the corresponding author upon request.
